# The Symptoms and Treatment of Rhus Poisoning

**Published:** 1892-02-27

**Authors:** 


### The Practitioner's Mirror and Retrospect.

[*The Editor will be glad to receive offers of co-operation and contributions from members of the profession. All letters should be addressed to* The Editor, The Lodge, Porchester Square, London, W.]

### MEDICINE.

### THE SYMPTOMS AND TREATMENT OF RHUS POISONING.

In this country poisoning by rhus is rare, but in some countries and localities it is far more common. The therapeutical properties of rhus toxicodendron were discussed in our issue of March 21st, 1891. Now that the tincture of various species of rhus are used as remedies, it is well that the symptoms of poisoning should be borne in mind, more especially as a pronounced idiosyncrasy is found in a few cases.

A case in point was recently reported by Mr. Arthur Prichard, of Bristol, in the *Bristol Medico Chirurgical Journal*, in which a serious attack of erysipelas was induced by contact with the juice of the rhus venenata, or "swamp sumach." The rhus venenata, belonging to the natural order anacardiaceæ, is a native of North America and Canada. It is a tall-growing shrub 15 or 20 feet high, with beautiful shining pinnate leaves. According to Gray's supplement to the *Pharmacopœia*, 1847, the juice, or even the air impregnated with the volatile principle of the plant, is to many persons a serious poison, producing severe and dangerous erysipelatous swellings. Khan mentions a person who, by the simple exhalation, was swollen to such a degree that he was as stiff as a log of wood, and could only be turned about in sheets. Another authority in the *Arboretum Britannicum*, says that the milky juice stains dark brown, and is poisonous to those who touch or smell it. In forty-eight hours inflammation appears in the skin in large blisters, principally in the extremities, attended with a burning and swelling; in two or three days the eruptions suppurate.

The case reported by Mr. Prichard was that of Mr. X. *aet.* 25, a curate having his study overshadowed by a fine specimen of the tree growing in the vicarage garden. He was persuaded to cut it down, but hearing that a gardener, three years before, had suffered from blood poisoning after cutting off a limb from the same tree, he started on the work with a certain amount of misgiving. He procured the help of the same gardener, but after the first afternoon's work the gardener fell ill, next day suffering from an attack of erysipelas, which kept him ill for a week. Mr. X. worked at the tree every day for a week, at the end of which time the tree came down. On October 23, some of the juice touched his face, and he wiped it off with his gloved hand. On October 24 he had three lines of redness on the cheek, which developed into an attack of facial erysipelas, and this grew persistently worse. On October 30 he was so ill that he had to keep to his bed; the arms, genitals, and thighs were attacked first by a red rash, then bullæ appeared, suppurated and burst, and large crusts formed, the exudation from under him being very profuse. On November 25th he was still in bed with the whole of his face, throat, and neck a mass of black scabs, some of which were partly loosened by the copious purulent exudation underneath them; he was unable to see on account of the stiffness of his closed lids, and unable to masticate from the same condition of his cheeks. On raising the lids pus poured from his eyes; the conjunctivæ were swollen, but the cornea bright and the sight good. The forearms and hands, the front and inside of the thighs, and the lower part of the abdomen were in somewhat the same condition as his face, covered with large crusts surrounded by a pink scab; but the crusts were not so dark as on his face. The effluvium emitted by the patient was foul and peculiar. He was very querulous, complaining most of the itching of his eyes, or the burning of his skin. During the next week he slowly improved. The treatment throughout the case was tonic and antiseptic. The observations of the older writers about this plant with regard to the susceptibility of the poison by individuals, are borne out in this case by the fact that this gentleman and the gardener were the only two who were affected by the poison, although several others joined in the work of cutting down, and were not influenced by the poison. In America these symptoms of rhus poisoning are fairly common, and the treatment should be well understood, but, as remarked by a leading American contemporary,* which we will quote *in extenso*, the many vaunted and specific cures persistently brought forward year after year as "almost of lightning rapidity in their effect," would seem to indicate that some enlightenment or reiteration is still called for on these points. It is, first of all, not to be lost sight of that the inflammatory action is of artificial origin, that it is produced by an accidental, short-acting, external irritant, and is not in any sense an idiopathic inflammation, such as eczema and similar diseases. Moreover, individuals differ as regards susceptibility, some probably being invulnerable to the poison, others but slightly susceptible; while in others, again, the action excited is extremely violent in character.

Rhus poisoning may be either mildly erythematous, erysipelatous, vesicular, pustular, or bullous. All these types will disappear spontaneously, some, owing to the degree of inflammation, taking longer for repair than others. The type has, therefore, much to do with the duration of the attack.

With the nature, behaviour, and self-limited character of the condition recognised, the choice of proper remedial measures is not a difficult one. They consist of protective, soothing, astringent, and antipruritic applications. There are numbers of such remedies, as literature will show, which may be used successfully, safely, and with comfort to the patient. For the erythematous type, with or without erysipelatous swelling, lotions and dusting powders are the most grateful. Among the former may be mentioned carbolic acid, in the strength of one or two drachms to the pint of water, to which may be added one to three drachms of alcohol and one-half to one drachm of glycerine; boric acid wash, fifteen grains to the ounce, with or without a minute quantity of glycerine, and, if the itching or burning requires it, a small quantity of carbolic acid; borophenol, five or ten grains to the ounce; ichthyol, one to five grains to the ounce; zinc sulphate, one-half to one drachm to the pint; and fluid extract of grindelia robusta, two to six drachms to the pint. The wash employed may often be advantageously followed by a dusting powder, such as rice flour, starch, boric acid, or talc, or a mixture of these; or, in some of the extremely mild cases, the powder used alone forms a clean and comfortable method of treatment. For the moderately severe type, with vesiculation, the same remedies and plans as outlined above may be likewise employed, and found all that is required; or, what is probably more satisfactory in these cases, the wash used may be supplemented with an ointment such as the oxide of zinc ointment, calamine, cold cream, or petroleum. Especially valuable is the combined treatment by lotio nigra and the oxide of zinc ointment. In this type also, in some instances, greater effect seems to follow the application of an ointment alone. In the severe pustular and bullous types of the inflammation, the methods above mentioned may also be used as stated, or with some modifications. If a wash is used alone, it should be applied by means of wrapping of linen or soft cotton, the parts being kept thus constantly and

* *Therapeutic Gazette*, Aug. 15, 1891.

thoroughly wet. Ointments, if employed, must be thickly applied, spread upon patent lint or similar material, a small quantity of carbolic acid, thymol, or resorcin added to control the burning or itching present.

